# The societal costs of chronic pain and its determinants: The case of Austria

**DOI:** 10.1371/journal.pone.0213889

**Published:** 2019-03-20

**Authors:** Susanne Mayer, Jonah Spickschen, K. Viktoria Stein, Richard Crevenna, Thomas E. Dorner, Judit Simon

**Affiliations:** 1 Department of Health Economics, Center for Public Health, Medical University of Vienna, Vienna, Austria; 2 Department of Social and Preventive Medicine, Center for Public Health, Medical University of Vienna, Vienna, Austria; 3 International Foundation for Integrated Care, Wolfson College, Oxford, United Kingdom; 4 Department of Physical Medicine, Rehabilitation and Occupational Medicine, Medical University of Vienna, Vienna, Austria; 5 Ludwig Boltzmann Institute Applied Diagnostics, Vienna, Austria; University of Nebraska Medical Center, UNITED STATES

## Abstract

**Background:**

Chronic pain is among the most burdensome conditions. Its prevalence ranges between 12% and 30% in Europe, with an estimated 21% among Austrian adults. The economic impact of chronic pain from a societal perspective, however, has not been sufficiently researched. This study aims to provide an estimate of the societal costs for working-age adults with chronic pain in Austria. It explores the impact of sex, number of pain sites, self-reported pain severity, health literacy and private health insurance on costs associated with chronic pain.

**Methods:**

A bottom-up cost-of-illness study was conducted based on data collected from 54 adult patients with chronic pain at three Viennese hospital outpatient departments. Information on healthcare costs including out-of-pocket expenses and productivity losses due to absenteeism and informal care were collected over 12 months. Resource use estimates were combined with unit costs and mean costs per patient were calculated in € for year 2016.

**Results:**

Mean annual societal costs were estimated at EUR 10191. Direct medical costs were EUR 5725 including EUR 1799 out-of-pocket expenses (mainly pain relieving activities and private therapy). Productivity losses including informal care amounted to EUR 4466. Total costs for women and patients with three or more pain sites were significantly higher. No association with health literacy was found but there was a tendency towards higher out-of-pocket expenses for patients with complementary private health insurance.

**Conclusion:**

This study is the first to provide a comprehensive assessment of the individual and societal burden of chronic pain in Austria. It highlights that chronic pain is associated with substantial direct medical costs and productivity losses. Patient costs may show systematic differences by health insurance status, implying a need for future research in this area.

## Introduction

Chronic pain is considered as one of the most burdensome diseases in industrialized countries [1]. According to the International Association for the Study of Pain (IASP), chronic pain is defined as “pain without apparent biological value that has persisted beyond the normal tissue healing time (usually taken to be 3 months)” [2]. Besides high costs for disease management, it is associated with major impacts on daily activities and quality of life [1] and high productivity losses due to work absences [3], partly due to common co-morbidities such as depression [4, 5]. In an early European survey from 2003 [3], the prevalence of chronic pain (defined as duration ≥6 months) was found to vary between 12% and 30% in 16 countries. It was highest amongst the age group of 41 to 60 years and higher among women than men. Available pain management was considered inadequate by 40% of the survey participants. Mean duration of chronic pain was 5.8 years. Most commonly it was caused by herniated/deteriorating discs, followed by traumatic injury and arthritis/osteoarthritis [4].

In the same study, prevalence of chronic pain was estimated at 21% for Austria, amounting to 1.83 million people in 2003 [4]. According to the Austrian health interview survey (ATHIS) 2006/2007, 39% suffered from substantial pain within the last 12 months of whom 64% experienced pain lasting longer than three months [6]. Most commonly, chronic pain was located in the spine area [6]. Diseases of the musculoskeletal system were the second most important contributor to all Austrian sick leave days in 2015 [7, 8]. In the 2014 ATHIS survey, 25% of all chronic pain patients reported being on pain-related sick leave in the past year [9]. Despite this high individual and societal impact, chronic pain is still often only considered a symptom instead of a major public health problem in its own right [10].

To assess the societal and economic impact of chronic pain, several cost-of-illness studies have been conducted across Europe (e.g. [11–17]). Differences in methodologies, costing approaches and populations, however, make comparisons across studies difficult. Most studies focus on direct medical costs with or without including broader societal costs such as productivity loss or informal care (e.g. [11–14, 17]). Although their inclusion is vital to reflect the societal burden comprehensively [1, 18], only a few previous studies (e.g. [15, 16]) captured medical costs that affected patients directly [19]. This is especially important in health care systems where co-payments or other out-of-pocket health expenses play a considerable role such as in Austria. In 2015, 26% of the total health expenditure was estimated to be private health spending [20]. Although 99.9% of the Austrian population is covered by the statutory social health insurance, 36% of the population also has complementary (voluntary) private health insurance (2014), resulting in a two-tier health care system. [21, 22].

Regarding drivers for differences in the costs of chronic pain, several studies have investigated associations between demographic factors, socioeconomic characteristics and disease severity with cost levels (e.g. [12, 15, 23–25]) to draw conclusions on the costs of different subgroups. However, little is known about the economic effect of health literacy on the costs of chronic pain. Health literacy is commonly defined as the ‘cognitive and social skills which determine the motivation and ability to gain access to, understand and use information in ways which promote and maintain good health’ [26]. Earlier research has shown that these skills affect the use and utilization pattern of health care services in general [27]. Therefore, it would be expected that differences in specific cost components (e.g. preventive care, emergency care, productivity losses) are related to differences in health literacy as well. The same applies to complementary private health insurance, which might affect both the uptake of health care services and the amount of out-of-pocket expenses for private medical treatment [16]. These hypotheses have been rather neglected by previous research. In addition, the societal burden of chronic pain has not yet been estimated in Austria.

The aim of this cost-of-illness study was to estimate the economic burden of chronic pain in working-age individuals from the health care, patient and societal perspectives. The impact of sex, number of pain sites, self-reported pain severity, health literacy and complementary private health insurance status on costs associated with chronic pain were also explored. Such a cost-of-illness analysis allows assessing the overall economic impact of chronic pain and its main cost components, aids the priority setting for health care policies and may inform future resource allocation [18, 28].

## Methods

### Data collection and sample

Patient recruitment took place between December 2012 and February 2014 as part of the study ‘Chronic pain and its development depending on social environment, health literacy, and previous treatment and associated costs’. Participants were recruited from three hospital outpatient departments (Clinic for Physical Medicine and Rehabilitation, Medical University of Vienna; Headache Outpatient Clinic, Medical University of Vienna; Pain Outpatient Department) located at two (publicly-funded, [29]) Viennese hospitals (General Hospital of Vienna; Orthopedic Hospital Speising). Due to the lack of an operative gate-keeping in the Austrian health care system [30] access to these outpatient departments is not restricted; no referral is needed and the consultations are free of charge. Therefore, no a-priori inference on the patient sample e.g. regarding disease severity can be drawn. Patient inclusion criteria were: i) chronic pain for three months or more (based on the screening criteria by Breivik and colleagues [3]), ii) between 18 and 65 years old, and iii) sufficient proficiency in German. Exclusion criteria were: i) current psychiatric inpatient stay and ii) patients suffering from cancer at the time of the study. 121 patients were eligible and initially consented to take part in the study.

Following a medical check, participants were informed about the study. After signing the informed consent form, participants completed the baseline questionnaire with the help of a research assistant. The baseline questionnaire captured socio-demographic details including sex, age, relationship status, employment status, highest educational attainment, monthly household income (after taxes), complementary private health insurance and exemption from prescription fees. The location and derived number of pain sites was assessed based on a pictorial representation. The number of pain sites was categorized into ‘1 or 2 pain sites’ and ‘3 or more pain sites’ and served as a proxy for the widespread nature of the pain. A visual analogue scale (VAS) ranging from 0 (indicating ‘no pain at all’) to 100 (‘the worst possible pain’) captured the subjective pain intensity and was categorized into high self-reported pain severity (VAS>36) and low pain severity (VAS≤36) based on the observed mean [31]. Polypharmacy was defined as using four or more prescription medicines within one quarter. Health literacy was assessed based on three items (adapted from an early self-reported screening instrument [32]): i) needing external help to read hospital material, ii) being confident in filling out medical forms alone, and iii) having problems learning about medical conditions because of difficulty with understanding written information. A total score was calculated with 3 points reflecting the worst possible health literacy level and 15 points reflecting the best possible health literacy level.

A cost diary was used to capture disease-attributable resource use within the past three months. It was informed by previous research [23] and piloted for the current study. At baseline, participants were trained by a research assistant in how to fill in the cost diaries, completed their baseline assessment and took home the follow-up diaries. Every three months, patients were contacted via phone to transmit the relevant follow-up data from their diaries. Alternatively, patients could hand-in the completed cost diaries in the outpatient department.

### Resource use and costs

Total costs were calculated from a societal perspective following a bottom-up approach [18]. Direct medical costs included all services covered and funded by the public health insurance scheme relevant for chronic pain patients, i.e. prescription medication, radiological procedures (X-ray, bone density measurement, magnetic resonance therapy, computer tomography, sonogram), inpatient stays, outpatient consultations including physiotherapy, as well as any out-of-pocket expenses for the patients. User charges for these services may arise depending on the individual’s statutory health insurance fund [33]. Study participants were asked to list these co-payments (including prescription fees) as out-of-pocket expenses. The same applies for services provided by non-contracted providers. Out-of-pocket expenses captured all relevant costs from the patient’s perspective and included over-the-counter (OTC) medication, expenses for prescription fees, household help, privately funded therapists (e.g. physiotherapists, homoeopathists, other therapists), pain related lifestyle activities and other disease-related out-of-pocket costs (e.g. for home adaptations, medical devices, co-payments). Depending on the complementary private health insurance plan that an individual may have and its form [33], some of these out-of-pocket expenses may eventually be covered under the patient’s private insurance scheme. This, however, was not formally assessed in the study. Societal costs additionally included productivity loss e.g. due to absence from work due to early retirement/disability, absence from work for medical treatments, sick leave and informal care provided by family members, friends or acquaintances. Presenteeism, i.e. working but work performance being impacted by ill health, was also measured based on a VAS with 0 indicating ‘not capable of working’ and 100 referring to the ‘best working capability’, but not expressed in monetary terms.

Resource use data were multiplied with unit cost estimates to calculate costs. Unit costs were retrieved from a number of sources (Table 1) based on the DHE Unit Cost Online Database for Austria [34, 35]. Direct medical services (excluding out-of-pocket expenses) were valued from the perspective of the public payer (for details, see S1 Text). Productivity loss was valued based on the average gross income in Austria using the human capital approach as recommended in the Austrian pharmacoeconomic methods guidelines [36] and most commonly used internationally [37]. Informal caregiving was valued at the level of minimum wage [38].

**Table 1:** Unit costs (in EUR for year 2016) by cost components.

Resource use	Unit cost (EUR)	Unit	Reference
**DIRECT MEDICAL COSTS**			
Prescription medication	5.20 (min.)-1037.70 (max.)	Per item	[40, 41]
X-ray	71.32	Per examination	[42]
Bone density measurement	27.73	Per examination	[43]
Magnetic resonance tomography (MRT)	728.06	Per examination	[44–46]
Computer tomography (CT)	248.10	Per examination	[47]
Sonogram	36.04	Per examination	[47]
Inpatient rehabilitation	200.03*	Per day	[39, 48]
Inpatient stay	762.00*	Per day	[39, 49]
Outpatient general practitioner (GP) in physician practice	11.92*	Per consultation	[39, 50]
Outpatient specialist in physician practice	46.34*	Per consultation	[39, 50]
Hospital outpatient department	91.78 61.19	Per first consultation Per further consultation	[51]
Physiotherapy	19.34	Per session	[52]
**Out-of-pocket expenses**			
Over-the-counter (OTC) medication	3.22 (min.)-51.55 (max.)*	Per item	[40]
Prescription fee	5.70	Per item	[53]
Private therapists	Various	Per 3 months	Self-reported information from cost diaries
Household help	Various	Per hours per week	Self-reported information from cost diaries
Pain reducing lifestyle activities	Various	Per 3 months	Self-reported information from cost diaries
Supporting devices	Various	Per 3 months	Self-reported information from cost diaries
Chronic pain-related information meetings/workshops	Various	Per 3 months	Self-reported information from cost diaries
Other (chronic pain-related) expenses	Various	Per 3 months	Self-reported information from cost diaries
**PRODUCTIVITY LOSS**			
Lost productivity (part time)	14.35*	Per hour	[39, 54]
Lost productivity (full time)	18.17*	Per hour	[39, 55]
Informal care	10.94*	Per hour	[39, 56]

*inflated based on the medical component of the consumer price index [39];

min. = minimum, max. = maximum; a more detailed description of the valuation of direct medical services is given in S1 Text.

Based on the information provided in the four cost diaries, costs were calculated for 12 months. In the case of missing cost diaries, we assumed that costs described in 3, 6 or 9 months were representative for the missing months and reported them for a 12-month period. All costs were calculated in Euro for year 2016 to reflect the latest available unit cost information at the time of manuscript submission. Cost data from previous years were inflated based on the national medical component of the consumer price index [39].

### Statistical analyses

The database was initially set-up in Microsoft Excel (2013) and double-checked by two authors (JSP, SM). Most cost diaries were fully completed. Missing items only accounted for 0.5% of all data entries and were replaced with mean values from all patients with relevant resource use. As some patients dropped out during the study, the cost data of these participants were extrapolated proportionately based on their available cost diaries. A sensitivity analysis restricted to patients with full cost information over the 12 months follow-up period was also conducted.

All statistical analyses were performed using Stata 14 [57]. Descriptive statistics of baseline characteristics are presented as means and standard deviations (SD) or percentages (%). Differences in baseline characteristics were explored via univariate linear regression analysis and Fisher’s exact test or Chi-square test where applicable. All cost analyses were initially conducted in a multivariate ordinary least square regression framework to determine statistically significant associations between (logged) costs and the variables of interest and included control variables such as age, sex and number of pain points (results not shown). Given the low number of observations, we instead report bivariate cost analyses by sex, number of pain sites, self-reported pain severity, health literacy and complementary health insurance status based on Wilcoxon rank sum tests. Two-sided p-values ≤0.05 were considered statistically significant. All costs are presented as mean, standard deviation (SD) and for aggregated direct medical, societal and total costs also as median.

The study was approved by the Ethics Committee of the Medical University of Vienna (No 1624/2012; preliminary approval: 04/09/2012, final approval: 03/04/2013) for the General Hospital of Vienna and the municipality of Vienna (EK-13-085-VK; approval: 03/06/2013) for the Orthopedic Hospital Speising. While advance information of potential study participants was started in December 2012, no primary data were collected prior to receiving formal ethical approval.

## Results

### Patient characteristics

Out of a total of 121 participants initially enrolled in the chronic pain study, 54 participants had at least 3 months of cost data (full 6 months data: 40 participants, full 9 months data: 36 participants) and were included in the cost-of-illness analysis (main analysis). Thirty-three patients had full 12 months cost data and were included in the sensitivity analysis. With one exception, no differences in demographic, socio-economic and clinical characteristics (duration of pain, subjective intensity of pain) were found between all initially enrolled patients (n = 121), the responders (n = 54) and the patients with 12 months cost data (n = 33). A higher percentage of the initially enrolled patients (73%, n = 88) reported living in a partnership compared to the 54 patients (63%, n = 34) included in the main analysis (p = 0.03). Table 2 presents the baseline patient characteristics for all three samples.

**Table 2:** Main sample patient characteristics at baseline (n = 54) compared to patients included in the sensitivity analysis (n = 33) and all initially enrolled participants (n = 121).

	Patients initially enrolled in the study	Patients with at least 3 months cost data (main analysis)	Patients with full 12 months cost data (sensitivity analysis)
	n = 121	n = 54	n = 33
Sex (%)			
Men	32 (26.5%)	12 (22.2%)	9 (27.3%)
Women	89 (73.6%)	42 (77.8%)	24 (72.7%)
Age in years, mean (SD)	48.8 (9.9)	49.6 (8.0)	51.1 (7.5)
Age range	23–65	30–64	32–64
Living in partnership (%)	88 (72.7%)*	34 (63.0%)	23 (69.7%)
Working status (%)			
Missing	1 (0.8%)	-	-
Unemployed	20 (16.5%)	7 (13.0%)	3 (9.1%)
Retired, in training, home-maker	30 (24.8%)	10 (18.5%)	7 (21.2%)
Working full-time	49 (40.5%)	27 (50.0%)	15 (45.5%)
Working part-time	19 (15.7%)	10 (18.5%)	8 (24.2%)
Highest education (%)			
Primary education	21 (17.4%)	10 (18.5%)	6 (18.2%)
High school with diploma/apprenticeship	74 (61.2%)	30 (55.6%)	20 (60.6%)
Tertiary education	26 (21.5%)	14 (26.0%)	7 (21.2%)
Household income per month (after taxes) (%)			
Missing	6 (5.0%)	2 (3.9%)	1 (3.1%)
<1000 EUR	7 (6.1%)	2 (3.9%)	1 (3.1%)
1001–1500 EUR	19 (16.5%)	9 (17.3%)	4 (12.5%)
1501–3000 EUR	47 (40.9%)	23 (44.2%)	4 (43.8%)
>3000 EUR	42 (36.5%)	18 (34.6%)	13 (40.6%)
Complementary private health insurance (%)	22 (18.2%)	9 (16.7%)	6 (18.2%)
Exempt from prescription fee (%)	10 (8.3%)	2 (3.7%)	1 (3.0%)
Duration of pain in months, mean (SD)	184.9 (173.4)	201.8 (187.8)	208.2 (186.5)
Number of pain points, mean (SD)	4.1 (2.8)	3.6 (2.5)	3.5 (2.3)
Location of pain (sorted by top 5, n = 121)^1^			
Pain in shoulders	70 (57.9%)	23 (54.8%)	17 (51.5%)
Neck pain	62 (51.2%)	24 (57.1%)	15 (45.5%)
Pain in knees, upper/lower legs	61 (50.4%)	15 (35.7%)	14 (42.4%)
Lower back pain	54 (44.6%)	19 (45.2%)	14 (42.4%)
Head (headache or migraine)	43 (35.5%)	17 (40.5%)	12 (36.4%)
VAS pain scale (0–100), mean (SD)	39.8 (25.8)	35.9 (24.4)	35.2 (23.2)
Health literacy			
Missing	1 (0.8%)	1 (1.9%)	1 (3.0%)
Score (3–15), mean (SD)	12.2 (2.3)	12.0 (2.3)	11.9 (2.5)

^1^As patients could report more than one pain site, numbers do not add up to 100%.

VAS = visual analogue scale with 0 indicating ‘no pain at all’ and 100 ‘the worst possible pain’; SD = standard deviation.

In total, 78% of the study sample were female (n = 42) (Table 2). Men and women were found to differ statistically significantly in terms of working status (with men being more likely to be employed, p = 0.01) and monthly household income after taxes (with men reporting higher incomes, p = 0.02). Men and women also differed in their reported number of pain sites (women: 4.0, SD 2.7; men: 2.3, SD: 1.4; p = 0.05).

On average, patients were aged 50 (SD: 8.0) years, 69% (n = 37) worked and 63% (n = 34) lived in a partnership (Table 2). Fifty-six percent (n = 30) reported having a high school diploma as highest educational attainment and with 44% (n = 23) participants most commonly had a household income between EUR 1501 and 3000. Overall, 17% (n = 9) of the study participants had a complementary private health insurance. The mean pain duration was 16.8 years (SD: 15.7) and mean subjective pain intensity was 35.9 (SD: 24.4) on the VAS scale (0–100). Patients reported an average of 3.6 (SD: 2.5) pain sites. The areas around the neck, shoulders, lower back, head as well as pain in the legs were most commonly mentioned as the locations of the chronic pain. The mean health literacy score was 12.0 (SD: 2.3) on a scale of 3 to 15.

### Direct medical costs

**Fig 1:**
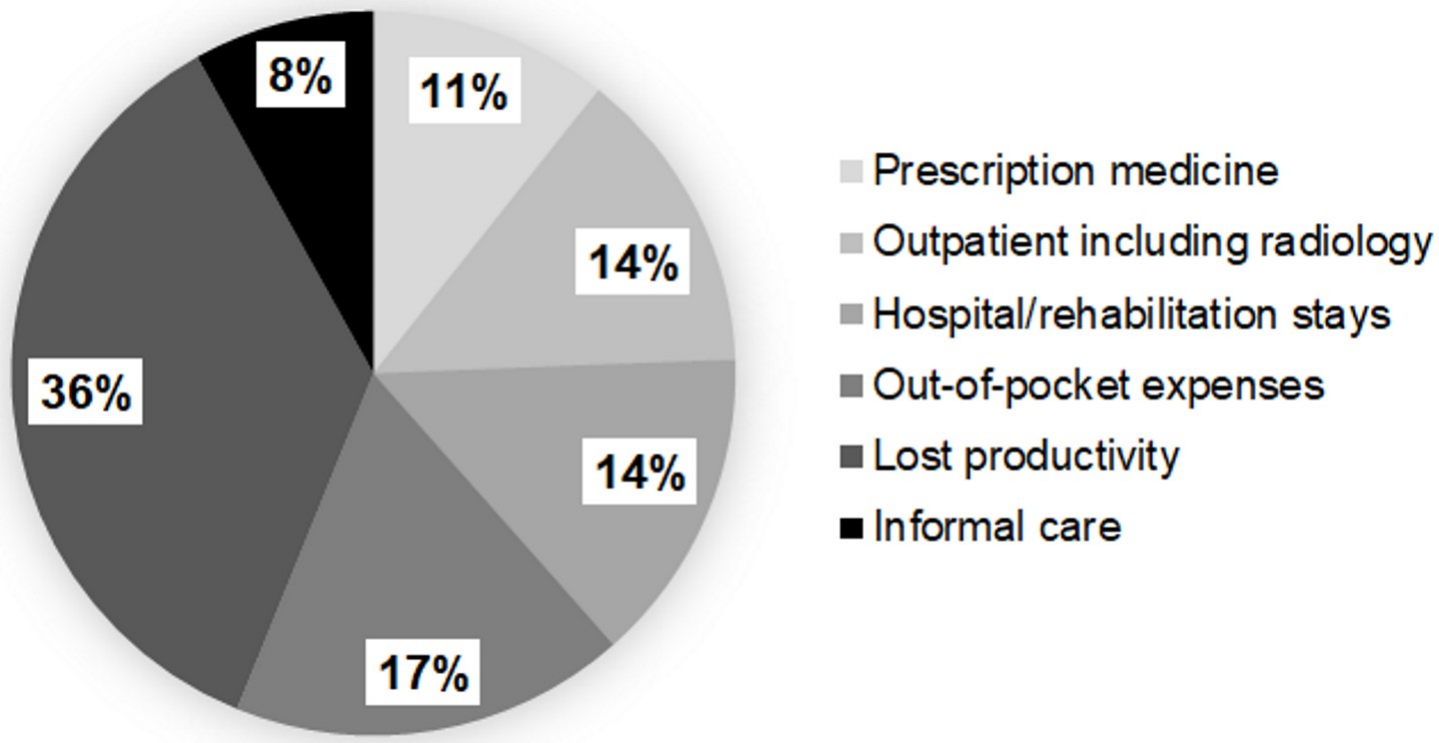
Societal costs by cost components as % of total costs (100%) (n = 54).

Details of the mean annual resource use are provided in Table 3 and of the mean annual costs in Table 4. Mean total direct medical costs (EUR 5725, SD: 6263, median: EUR 3782) contributed to 56% of total costs (Table 4, Fig 1). Direct medical costs excluding out-of-pocket expenses contributed to 39% of mean total costs and amounted to EUR 3926 (SD: 5448, median: EUR 1334). Inpatient costs (especially hospital stays) (EUR 1436, SD: 2813, 14%), followed by prescription medication (EUR 1092, SD 3965, 11%) and outpatient costs (especially physiotherapy costs) (EUR 849, SD 894, 8%) contributed the highest costs from the perspective of the public payer. In terms of resource use, GP visits were the most common (63%, n = 34), followed by specialist consultations (57%, n = 31) and physiotherapist sessions (52%, n = 28) (Table 3).

**Table 3:** Outpatient and inpatient resource use (n = 54) and productivity loss (n = 45) over 12 months.

	n* (patients)	% (patients with resource use)	Mean per patient with resource use	Mean resource use all patients	SD
**HEALTH CARE USE**	**All (n = 54)**				
**Outpatient resource use (unit)**					
GP in physician practice (consultations)	34	63.0	11.4	7.2	9.1
Specialist in physician practice (consultations)	31	57.4	9.0	5.2	8.8
Hospital outpatient department (consultations)	17	31.5	7.5	2.4	5.3
Physiotherapy (consultations)	28	51.9	36.4	18.9	28.5
**Inpatient resource use**					
Inpatient rehabilitation (days)	9	16.7	27.0	4.5	10.8
Inpatient hospitalization (days)	4	7.4	9.5	0.7	2.7
**PRODUCTIVITY LOSS (unit)**	**All (n = 45)**^+^				
Due to disability pension or part-time work (days)	4	8.9	159.6	14.2	49.0
Due to physician consultations (hours)	17	37.8	26.2	8.8	23.8
Due to sick leave (days)	11	24.4	64.0	15.9	56.3
Due to rehabilitation or inpatient stay (days)	9	20.0	24.0	4.4	11.0
Impaired productivity at work (0–100)	45	100.0	63.0	63.0	30.0

*n = number of patients with at least one 1 unit of resource use;

GP = general practitioner; SD = standard deviation;

^+^lost productivity reported for patients in employment (n = 45).

**Table 4:** Observed mean annual costs per patient with chronic pain (in EUR for year 2016): Main analysis (n = 54) and sensitivity analysis (n = 33).

	Main analysis (n = 54)				Sensitivity analysis (n = 33)			
Cost component	n (patients) with costs >0	Mean costs in EUR	SD	% of total costs	n (patients) with costs >0	Mean costs in EUR	SD	% of total costs
**DIRECT MEDICAL COSTS**	52	5725	6263	56.2	33	5327	4837	60.0
**Prescription medication**	33	1092	3965	10.7	23	1012	3562	11.4
**Radiological procedures**	24	548	1466	5.4	16	257	499	2.9
**Outpatient costs**	47	849	894	8.3	30	751	774	8.5
GP in physician practice	34	86	109	0.8	25	94	98	1.1
Specialist in physician practice	31	240	409	2.4	23	205	263	2.3
Hospital outpatient department	17	158	338	1.6	12	135	233	1.5
Physiotherapy	28	365	552	3.6	18	317	525	3.6
**Inpatient costs**	13	1436	2813	14.1	10	1805	3085	20.3
Inpatient rehabilitation	9	900	2162	8.8	6	927	2125	10.4
Inpatient stay	4	536	2054	5.3	4	878	2585	9.9
**Out-of-pocket costs**	48	1799	2683	17.7	32	1502	1330	16.9
Over-the-counter (OTC) medication	32	82	123	0.8	22	97	138	1.1
Prescription fee	31	51	85	0.5	22	58	94	0.7
Private therapists	23	425	843	4.2	16	426	741	4.8
Household help	11	381	897	3.7	6	322	789	3.6
Pain reducing lifestyle activities	27	661	1659	6.5	18	391	573	4.4
Other expenses	26	198	435	1.9	20	208	313	2.3
**PRODUCTIVITY LOSS**	35	4466	9212	43.8	23	3545	7167	40.0
Lost productivity due to early retirement/disability pension or part-time work	4	1641	6348	16.1	2	1115	4535	12.6
Lost productivity due to physician consultations	17	138	366	1.4	12	124	342	1.4
Lost productivity due to sick leave	11	1411	5630	13.8	7	1033	3470	11.6
Lost productivity due to rehabilitation stays	7	406	1216	4.0	5	346	916	3.9
Lost productivity due to inpatient hospitalization	2	49	288	0.5	2	80	368	0.9
Informal care	18	823	1890	8.1	12	849	2033	9.6
**TOTAL COSTS**	53	10191	12472	100.0	33	8871	10106	100.0

GP = general practitioner; SD = standard deviation.

Out of the 54 patients, 33 (61%) reported having taken at least one prescription medication over the last 12 months (Table 4) and 15% of these patients (n = 5) took 4 or more prescribed pharmaceuticals. Adjuvant pain medication (i.e. pharmaceuticals that have an indirect positive effect on the pain therapy or treat the underlying disease causing the chronic pain, see S2 Text for the list of included medications) was the main contributor (95%) to the cost of prescription medications. With regards to the type of prescription pain medication, 91% of the patients (n = 30) took at least one non-steroid anti-inflammatory drug (NSAID), 70% (n = 23) at least one adjuvant pain medication, 12% (n = 4) at least one opiate and 12% (n = 4) at least one other type of prescription pain medication.

From the viewpoint of the patient, pain reducing lifestyle activities that prevent or alleviate chronic pain (e.g. sports like swimming and yoga) (EUR 661, SD: 1659, 7%), private therapist costs (EUR 425, SD: 843, 4%) and household help (EUR 381, SD: 897, 4%) were the dominant contributors to out-of-pocket expenses (Table 4). A total of 32 patients (60%) reported taking OTC medication, amounting to annual costs of EUR 82 (SD: 123, 0.8%). Almost all patients (n = 48, 89%) reported incurring some expenses related to their chronic pain condition, resulting in an average of EUR 1799 (SD: 2683, median: EUR 1003, 18%) out-of-pocket costs per year.

### Societal costs

Overall, productivity losses (EUR 4466, SD: 9212, median: EUR 1019, 44%) contributed to almost half of the total costs (Table 4, Fig 1). Productivity loss due to early retirement/disability pension or part-time work was the main contributor (EUR 1641, SD: 6348, 16%). Four patients reported that they had to switch from working full-time to part-time (n = 2, 4%) or had to go into disability pension (n = 2, 4%) due to their chronic pain. This corresponded to losing an average of 159.6 days of work (SD: 65.4) (Table 3). All four patients reported widespread pain (4–5 pain sites). The second highest productivity losses were associated with sick leave (EUR 1411, SD: 5630, 14%) (Table 4). The mean number of sick days was estimated at 15.9 (SD: 56.3) per patient in employment (n = 45) (Table 3). All of these patients stated that they had already attended work despite being in severe pain with a self-reported mean productivity of 63 (SD: 30) on the VAS scale. Informal care incurred EUR 823 (SD: 1890, 9%) in productivity loss costs (Table 4).

Mean total annual cost per chronic pain patient were estimated at EUR 10191 (SD 12472, median: EUR 5145) (Table 4). In the sensitivity analysis based on full 12 months cost data, overall mean costs were found reduced by EUR 1320 (-13%). This reduction was mostly driven by the differences in lost productivity costs (Δ EUR 921, -21%). Median total costs were found to be more similar (EUR 4985, n = 33) and the patterns of cost distribution were also comparable in both analyses (Table 4). In the main analysis, the top 5% of patients with the highest total costs were found to consume 25% of overall costs, which emphasizes the high skewness of the cost data.

### Determinants of cost differences

Total costs for men and women differed significantly (Δ EUR 9124, p = 0.02) and were estimated at EUR 3095 (SD: 2709) and EUR 12219 (SD: 13422), respectively (Table 5). Mean total direct medical costs were statistically significantly higher for women than men (Δ EUR 4318, p = 0.04) and there was a tendency for women to spend more out-of-pocket (Δ EUR 1489, p = 0.06). Another clear difference in costs could be observed by the number of pain sites. For example, overall direct medical costs (Δ EUR 3815, p = 0.01), outpatient costs (Δ EUR 396, p = 0.01), inpatient rehabilitation costs (Δ EUR 1365, p = 0.02), out-of-pocket expenses (Δ EUR 1119, p = 0.02) and productivity losses (Δ EUR 2908, p = 0.01) were found to be higher for people with three or more pain sites. In line with this, mean total costs were also higher for patients with a higher number of pain sites (Δ EUR 6723, p<0.001). On the other hand, costs were not statistically significantly associated with self-reported pain severity (results not shown). The same applies to health literacy, for which no statistically significant differences were detected (results not shown). Finally, direct medical costs, lost productivity and total costs were not found to be statistically different by health insurance status (results not shown). However, there was a tendency for total out-of-pocket costs to be higher for people with a complementary private health insurance (Δ EUR 903, p = 0.09). This finding is partially driven by the higher private therapy costs for those with private complementary health insurance (Δ EUR 126, p = 0.11).

**Table 5:** Observed mean annual costs per patient with chronic pain by sex and number of pain points (in EUR for year 2016) (n = 54).

	Men (n = 12)		Women (n = 42)			1 or 2 pain points (n = 24)		3 or more pain points (n = 30)		
Cost component	Mean in EUR	SD	Mean in EUR	SD	P-value	Mean in EUR	SD	Mean in EUR	SD	P-value
**DIRECT MEDICAL COSTS**	2366	1967	6684	6739	0.04	3605	4241	7420	7122	0.01
Prescription medication	428	972	1282	4461	0.74	218	701	1791	5216	0.13
Radiological procedures	219	513	642	1633	0.99	678	2009	445	839	0.74
Outpatient costs	457	450	961	960	0.12	629	958	1025	813	0.01
Inpatient costs	621	1478	1669	3065	0.37	904	2259	1863	3161	0.21
Out-of-pocket costs	641	556	2130	2952	0.06	1177	1969	2296	3082	0.01
**PRODUCTIVITY LOSS**	729	955	5534	10208	0.13	2851	9078	5759	9265	0.01
**TOTAL COSTS**	3095	2709	12219	13422	0.02	6456	12038	13179	12188	<0.001

SD = standard deviation.

## Discussion

This cost-of-illness study aimed to comprehensively assess the economic burden of chronic pain in the working-age population in Austria. Based on a sample of patients recruited from three hospital outpatient departments, annual societal costs were estimated at EUR 10191 per patient for the year 2016. The findings emphasize the high economic impact of chronic pain on both the individuals and the society. The highest direct medical costs were associated with out-of-pocket expenses (18%), inpatient costs (14%) and outpatient costs (8%). Highest productivity losses were associated with early retirement/work reductions (16%) and sick leave (14%). In terms of potential drivers for differences in chronic pain costs, women and patients with three or more pain sites had significantly higher total costs than men and patients with one or two pain sites. No association between costs and health literacy or self-reported pain severity was found. There was a tendency towards higher out-of-pocket expenses for chronic pain patients with a complementary private health insurance.

In comparison with another Austrian study measuring the direct costs of chronic low back pain in 2008 (EUR 1837 in 2008) [11], total direct medical costs were considerably higher in this study. Despite methodological discrepancies between the two analyses, inpatient rehabilitation and out-of-pocket costs were also found to be the most expensive cost factor in this earlier study [11], which is consistent with our findings. Compared to our study, total costs from the societal perspective were found to be lower in a Portuguese study (EUR 1883 in 2010) and in a Swedish study (EUR 6429 in 2008) [12, 14]. Both studies [12, 14] focused on wider chronic pain populations and did not consider out-of-pocket expenses. An Irish study drawing on a community-based sample of chronic pain patients also found a lower total cost estimate (EUR 5665 in 2008) [15]. While the study included some out-of-pocket expenses, it excluded e.g. private medical treatment costs. Another Irish cost-of-illness analysis of chronic pain patients attending specialized pain management clinics [16] compares more closely with our study, both regarding study population and cost components. This Irish study estimated the societal burden at USD 24043 in 2008 [16], which is considerably higher than our estimates. This discrepancy in findings may be related to differences in resource use, unit costs and health care systems. For example, both inpatient and hospital outpatient services were seemingly used more frequently by Irish patients and also the relevant unit costs were considerably higher [16]. Overall, however, international comparisons are generally impaired by a variety of cross-country differences affecting robust conclusions based on the relevant cost estimates [58].

On the other hand, the patterns in costs components found in our cost-of-illness analysis are consistent with other national and international studies. Out-of-pocket expenses were similar to the out-of-pocket expenses of Dutch chronic pain patients (USD 1350 in 1999) [59]. The tendency of higher out-of-pocket costs of women compared to men may be related to the higher number of pain sites reported by the female study population. At the same time, the higher expenses for pain reducing lifestyle activities are also in line with research highlighting gender differences in health promoting activities [60]. Polypharmacy, i.e. use of four prescription medications or more, was only present in five patients (15%). Although relevant definitions are not fully comparable across studies, this number seems low even in comparison to the general Austrian population (21%) [61]. Also in light of relevant international estimates [5], it seems possible that prescription costs are underestimated in our study due to the small sample size. Physiotherapy was the highest cost associated with outpatient costs (EUR 365). With EUR 425 an even higher amount was spent on private therapists, which points out the high relevance of this additional pillar of care for Austrian chronic pain patients. Productivity losses due to disability pension or having to work part-time instead of full time because of chronic pain were reported by four patients (7%). This proportion is smaller than the estimate derived in an Austrian survey of chronic pain patients of all age groups (21%) [62]. The observed annual average of 15.9 work days lost due to sickness is in line with an early estimate of 7.5 days over six months [3]. In comparison to the general Austrian population (12.3 sick work days in 2014) [63], chronic pain results in 3.6 additional sick days per year. These findings are in accordance with a Swedish study identifying sick leave and early retirement to be the main contributor to total costs [14] and point at the considerable societal cost savings that could potentially result from more effective management of pain [64, 65]. Total productivity losses generated almost half of the societal costs, which highlights the vast societal burden of chronic pain. Presenteeism was assessed (work productivity: 63%) but not included in our cost analysis. It was found to be below the estimate from a Swedish analysis on chronic low back pain (71%) [23] but higher than in a Danish study on chronic pain patients (51%) [17]. Overall, the ratio between direct medical costs and productivity losses found in our study is comparable to several international findings [12, 13, 15, 16, 25].

In terms of differences in chronic pain costs by subgroups, female sex was found to be a major determinant of cost differences. While this result is in line with some previous work [12, 23], it is also likely related to the gender difference in the baseline number of pain sites in our sample. Reporting three or more pain sites was associated with two-times higher mean costs, which is consistent with previous research from Ireland [15]. No differences in costs, however, were found by level of health literacy. This may be expected in light of the scope of the applied (early) screening instrument [32, 66, 67]. For example, sufficient proficiency in German was one of the study inclusion criteria, which might also explain the high average health literacy score in our sample. In contrast, a European study assessed health literacy in Austria to be generally low in international comparison [68]. Overall, previous international work highlighted the significant relation between suboptimal health literacy in patients with chronic conditions, poorer knowledge about the condition and reduced self-management capabilities [69]. A Portuguese study identified a trend towards higher costs for chronic pain patients with lower educational level, i.e. a factor that is likely to be highly correlated with health literacy [12]. Similarly to a low socioeconomic status, limited health literacy could also translate into a higher use of acute care services like emergency care [61], lower uptake of preventive care [70] or extended work absenteeism for chronic pain patients in Austria. Given that international research finds individuals with low self-care skills exposed to a higher risk of chronic diseases and also being more impaired by these conditions [71], this patient group is also of special interest from a public health perspective.

The identified tendency for higher out-of-pocket expenses for people with complementary private health insurance (especially when it comes to private therapy costs) needs to be interpreted in the light of the Austrian two-tier health system. While private health insurance status is likely to be also related to higher socioeconomic status [72], it is also expected that patients with a complementary health insurance are more likely to opt for a private alternative instead of facing the waiting times and possible lower levels of comfort components in the public sector [73, 74]. Depending on the individual health insurance contract and its coverage (e.g. hospital sector, outpatient sector), these additional costs that need to be first covered out of pocket may then be reimbursed by the private insurance. Effectively, this implies that patients with a private health insurance have higher resource use. Eventually, however, they may end up having lower service-related out-of-pocket expenses as these costs may ultimately be covered by the private insurance. This interpretation is in line with an Irish study that found total direct medical costs to be higher for chronic pain patients with a private health insurance [16]. As these authors did not explore this issue further and the validity of our finding is impaired by the small sample size, future cost-of-illness studies should consider testing if such a tendency also holds in similar health care systems.

### Strengths and limitations

To our knowledge, this is the first study to estimate the societal burden of chronic pain in Austria. In addition, this is one of the few studies internationally that measures related societal costs comprehensively. Besides investigating commonly researched associations between costs and demographic and clinical characteristics, this study also adds to the literature through the analyses of the impact of health literacy and complementary health insurance on the costs of chronic pain.

The findings of this cost-of-illness analysis need to be interpreted in the health care system context and considering potential limitations. The small study sample and recruitment from three outpatient clinics in the capital might impair the generalizability of the results to the national level. Firstly, with an average of 17 years, the duration of chronic pain was longer than the (more conservative) general Austrian estimate of 6 years from 2003 [3]. In addition, a recent German study also showed that only one in three patients with chronic pain are treated by pain specialists located in hospital outpatient departments [75]. Another earlier study points at a lower number in Austria as well [3]. Secondly, patient recruitment took place in one urban area characterized e.g. by an extensive medical infrastructure [76]. The patient sample was also slightly more educated than the general Austrian population [77], which is in line with previous findings that highly educated Austrians are more likely to (directly) consult specialists in hospital outpatient departments without a prior primary care contact [30]. Thirdly, the number of included patients is small and in some cases, only very few study participants contributed to some cost categories (e.g. inpatient stays). Together with the fairly short real follow-up time in some cases, this contributes to the uncertainty around the derived estimates. Compared to the 121 participants initially enrolled in the study, there were no significant differences in socio-demographic and clinical characteristics. This finding implies that overall costs might have been similar for the originally recruited study sample. Finally, another limitation stems from the fact that although the cost-of-illness study was conducted from a societal perspective, not all unit costs used in the analysis necessarily capture ‘social opportunity costs’ [78]. The used costing sources have to be seen as a pragmatic and currently the only feasible approach in Austria.

## Conclusion

The costs of chronic pain including patients’ out-of-pocket costs were found to be substantial in Austria and the trend towards systematic differences by health insurance status calls for future research in this area. The comparatively low proportion of patients contributing to the high productivity losses illustrates the need to emphasize and strengthen early diagnosis, prevention and better management of chronic pain as a priority from a public health policy perspective. More effective and efficient treatment programs–even if these treatment costs are high–could potentially reduce early retirement and sick days and lead to a good value policy option with overall lower cost-of-illness in Austria.

## Supporting information

S1 Text Valuation of direct medical services. (DOCX)

S2 Text Adjuvant pain medication. (DOCX)

S3 Text Dataset description. (DOCX)

S1 File Dataset chronic pain. (XLSX)
