# Supplementary material for: The societal costs of chronic pain and its determinants: The case of Austria
Source: PLoS One. 2019 Mar 20;14(3):e0213889. doi: 10.1371/journal.pone.0213889 (PMC6426226; doi:10.1371/journal.pone.0213889)
Supplement: S1 Text — (DOCX) [file pone.0213889.s001.docx]

**S1 Text. Valuation of direct medical services**

Prescription medication was valued at the prices covered by the public health insurance funds (‘Kassenverkaufspreise’) and prescription fees were considered out-of-pocket costs. For radiological procedures, hospital outpatient department consultations and physiotherapy sessions, fees legally set for billing services not covered through public funds were used. Costs per inpatient day were based on fees capturing the charges to non-insured patients per care day, while inpatient rehabilitation costs per day were specifically calculated based on the costs incurred by the Viennese Health Insurance Fund. Reimbursement data provided in the annual report by the Viennese Health Insurance Fund complemented with internal data from the Main Association of Social Security Institutions were used to calculate the costs of general practitioner (GP) visits and specialist consultations. Pharmacy prices (‘Apothekenverkaufspreise’) were used for over-the-counter (OTC) medication and any prescription medication priced below the prescription fee. All other out-of-pocket expenses, including therapist costs not covered by the social health insurance, were directly taken from the cost diaries.
