# Supplementary material for: The societal costs of chronic pain and its determinants: The case of Austria
Source: PLoS One. 2019 Mar 20;14(3):e0213889. doi: 10.1371/journal.pone.0213889 (PMC6426226; doi:10.1371/journal.pone.0213889)
Supplement: S2 Text — (DOCX) [file pone.0213889.s002.docx]

**S2 Text. Adjuvant pain medication**

- Beloc
- Convulex
- Ebetrexat
- Enbrel injection
- Humira
- Inderal
- Lyrika
- Neurobion
- Nomexor
- Pantoloc
- Prednisolon
- Relpax
- Saroten
- Sirdalud
- Topilex
- Tregetol
- Venlafaxin
- Xefo
- Zomig
- Zomig nose spray
