# Supplementary material for: The societal costs of chronic pain and its determinants: The case of Austria
Source: PLoS One. 2019 Mar 20;14(3):e0213889. doi: 10.1371/journal.pone.0213889 (PMC6426226; doi:10.1371/journal.pone.0213889)
Supplement: S3 Text — (DOCX) [file pone.0213889.s003.docx]

**Dataset**

**Variable description for file “dataset_chronic_pain”**

| **Variable name** | **Description** |
| --- | --- |
| nrcostdiaries | Number of available cost diaries, 1=3 months, 2=6 months, 3=9 months, 4=12 months |
| premed | Prescribed medication (excluding prescription fee, which is an out-of-pocket expense), in EUR |
| xray | Costs of x-rays, in EUR |
| bonedens | Costs of bone density measurements, in EUR |
| MRT | Costs of magnetic resonance tomography (MRT), in EUR |
| CT | Costs of computer tomography (CT), in EUR |
| sonogram | Costs of sonograms, in EUR |
| t_radio | Total costs of radiological procedures (xray + bonedens + MRT + CT + sonogram), in EUR |
| GP | Costs of general practitioner (GP) consultations in physician practices, in EUR |
| opspec | Costs of outpatient specialist consultations in physician practices, in EUR |
| outpatdep | Costs of hospital outpatient department consultations, in EUR |
| physiotherap | Costs of physiotherapy sessions, in EUR |
| t_outp | Total costs of outpatient treatments (GP + opspec + outpatdep + physiotherapy), in EUR |
| t_radiooutp | Total costs of outpatient treatments including radiological procedures (t_outp + t_radio), in EUR |
| rehab | Costs of inpatient rehabilitation stays, in EUR |
| inpatient | Costs of inpatient stays, in EUR |
| t_inpat | Total costs of inpatient stays (rehab + inpatient), in EUR |
| t_healthcare | Total health care costs (premed + t_radio + t_outp + t_inpat), in EUR |
| OTC | Costs of over-the-counter (OTC) medication, in EUR |
| prefee | Prescription fees, in EUR |
| privther | Costs of private therapists, in EUR |
| househelp | Costs of household help, in EUR |
| healthmeasures | Costs of pain reducing lifestyle activities, in EUR |
| suppdev | Costs of supporting devices, in EUR |
| events | Costs of chronic pain-related information meetings/workshops, in EUR |
| otherexpenses | Other (chronic pain-related) expenses, in EUR |
| t_otherexpenses | Total costs of other pain-related activities (suppdev + events + otherexpenses), in EUR |
| t_oop | Total out-of-pocket expenses (OTC + prefee + privther + househelp + healthmeasures + suppdev + events + otherexpenses), in EUR |
| t_healthoop | Total health care costs and out-of-pocket expenses (t_healthcare + t_oop), in EUR |
| lp_other | Lost productivity due to disability pension or part-time work, in EUR |
| lp_treatment | Lost productivity due to physician consultations, in EUR |
| lp_sick | Lost productivity due to sick leave, in EUR |
| lp_rehab | Lost productivity due to rehabilitation stays, in EUR |
| lp_inpat | Lost productivity due to inpatient stays, in EUR |
| t_lpwork | Total lost productivity (of the patient – excluding lost productivity related to informal care) (lp_other + lp_treatment + lp_sick + lp_rehab + lp_inpat), in EUR |
| informalcare | Lost productivity due to informal care by family members, friends or acquaintances, in EUR |
| t_lostprod | Total lost productivity including informal care (t_lpwork + informalcare), in EUR |
| totalcosts | Total health care costs including out-of-pocket expenses and lost productivity (t_healthoop + t_lostprod), in EUR |
| painpoints | Number of total pain points |
| sex | Sex (1=men, 2=woman) |
| HL_score | Health literacy score, 3-12 |
| VASpain | Visual analogous scale (VAS) pain scale, 0-100 |
| privHI | Private (complementary) health insurance (1=yes, 2=no) |
| paininmo | Chronic pain in months |
